# Surveillance, contact tracing and characteristics of SARS-CoV-2 transmission in educational settings in Northern Italy, September 2020 to April 2021

**DOI:** 10.1371/journal.pone.0275667

**Published:** 2022-10-10

**Authors:** Olivera Djuric, Elisabetta Larosa, Mariateresa Cassinadri, Silvia Cilloni, Eufemia Bisaccia, Davide Pepe, Massimo Vicentini, Francesco Venturelli, Laura Bonvicini, Paolo Giorgi Rossi, Patrizio Pezzotti, Alberto Mateo Urdiales, Emanuela Bedeschi, The Reggio Emilia Covid-19 Working Group

**Affiliations:** 1 Epidemiology Unit, Azienda Unità Sanitaria Locale–IRCCS di Reggio Emilia, Reggio Emilia, Italy; 2 Department of Biomedical, Metabolic and Neural Sciences, Centre for Environmental, Nutritional and Genetic Epidemiology (CREAGEN), Public Health Unit, University of Modena and Reggio Emilia, Reggio Emilia, Italy; 3 Public Health Unit, Azienda Unità Sanitaria Locale–IRCCS di Reggio Emilia, Reggio Emilia, Italy; 4 Department of Infectious Diseases, Istituto Superiore di Sanità, Rome, Italy; Health Directorate, LUXEMBOURG

## Abstract

**Background:**

The role of school contacts in the spread of the virus and the effectiveness of school closures in controlling the epidemic is still debated. We aimed to quantify the risk of transmission of SARS-CoV-2 in the school setting by type of school, characteristics of the index case and calendar period in the Province of Reggio Emilia (RE), Italy. The secondary aim was to estimate the speed of implementation of contact tracing.

**Methods:**

A population-based analysis of surveillance data on all COVID-19 cases occurring in RE, Italy, from 1 September 2020, to 4 April 2021, for which a school contact and/or exposure was suspected. An indicator of the delay in contact tracing was calculated as the time elapsed since the index case was determined to be positive and the date on which the swab test for classmates was scheduled (or most were scheduled).

**Results:**

Overall, 30,184 and 13,608 contacts among classmates and teachers/staff, respectively, were identified and were recommended for testing, and 43,214 (98.7%) underwent the test. Secondary transmission occurred in about 40% of the investigated classes, and the overall secondary case attack rate was 4%. This rate was slightly higher when the index case was a teacher but with almost no differences by type of school, and was stable during the study period. Speed of implementation of contact tracing increased during the study period, with the time from index case identification to testing of contacts being reduced from seven to three days. The ability to identify the possible source of infection in the index case also increased.

**Conclusions:**

Despite the spread of the Alpha variant during the study period in RE, the secondary case attack rate remained stable from school reopening in September 2020 until the beginning of April 2021.

## Introduction

Since the first case of COVID-19 was described at the end of 2019, there have been almost 254.1 million confirmed cases and 5 million deaths reported globally up to 14 November 2021 [1], but the estimated number of people who have been infected is about 3.4 billion [2]. Closure of educational institutions was one of the preventive measures considered and often adopted during the pandemic. This was due to the concern about potential school-to-home transmission of the virus from students to more susceptible family members, although the overall risk of severe COVID-19 in children and young people was shown to be very low [3].

The role of school contacts in the spread of the virus and the effectiveness of school closures in controlling the epidemic have been debated [4,5]. Modelling studies have provided scenarios showing a limited impact of school closures on virus transmission under the conditions hypothesized by the authors [6–8]. A recent systematic review of empirical studies showed heterogenous results ranging from no effect to a significant reduction in virus transmission in the community [9], with studies with lower risk of bias showing a limited effect [10,11]. Finally, three large studies from the USA, the UK and Italy suggest a limited role of school transmission in determining mortality in the community [12–14]. It is worth noting that school opening may impact virus transmission not only through contacts occurring in the classroom but also because of the increase in public transport-related contacts and other non-school activities.

**Fig 1:**
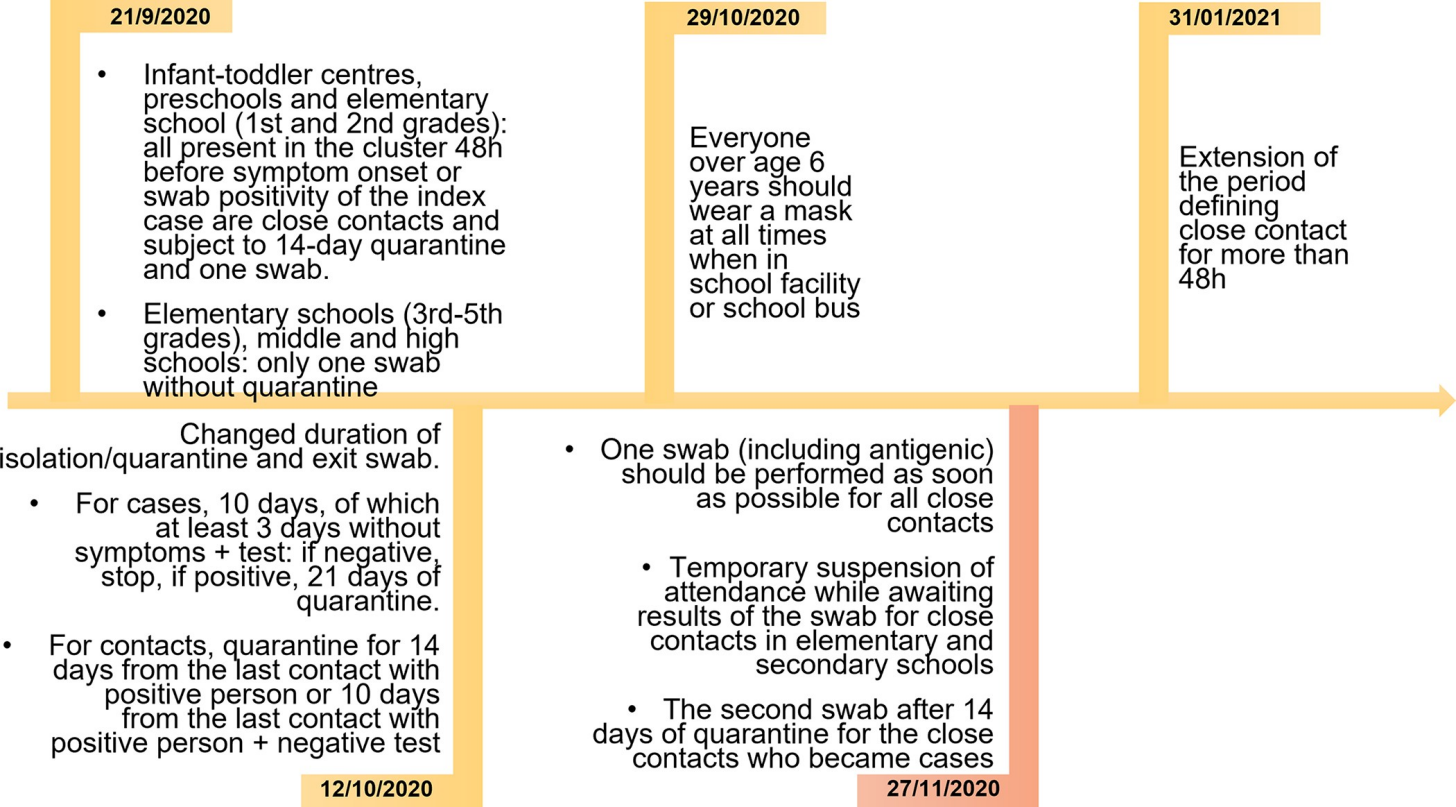
Timeline of the major changes in regulations and recommendations for management of COVID-19 cases in educational settings in Reggio Emilia.

In our first study conducted between 1 September and 15 October 2020, non-negligible secondary attack rates were detected, especially in secondary schools. After this first report, several changes occurred that could have influenced the risk of transmission and its control in schools [15]. Starting from 27 November 2020, the local health authority improved contact tracing protocols and introduced immediate molecular tests for all contacts–whether symptomatic or asymptomatic–at the beginning of quarantine, with the aim of identifying all possible sources of infection in asymptomatic contacts (in other words, backward tracing) (Fig 1). Policies to reduce crowding, especially in high schools, were introduced (reducing in-class time by 25% to 50%), as were several short closures in the periods of highest incidence. Finally, at the end of December 2020, the Alpha variant started to circulate within the province, becoming predominant in February 2021 [16].

In this study we present the results of comprehensive epidemiological investigations conducted from school reopening in September 2020 until the end of March 2021. Our aim was to quantify the risk of infection transmission in the school setting by type of school, epidemiological characteristics of the index case and calendar period in the Province of Reggio Emilia by analysing surveillance data about all cases who had contacts in the school setting. The secondary aim was to estimate the speed of implementation of contact tracing.

## Materials and methods

### Ethics statement

The study was approved by the Area Vasta Emilia Nord Ethics Committee (no. 2020/0045199). The Ethics Committee authorised the use of subject data even in the absence of consent for people that could not be reached if every reasonable effort had been made to contact that subject.

### Setting

**Fig 2:**
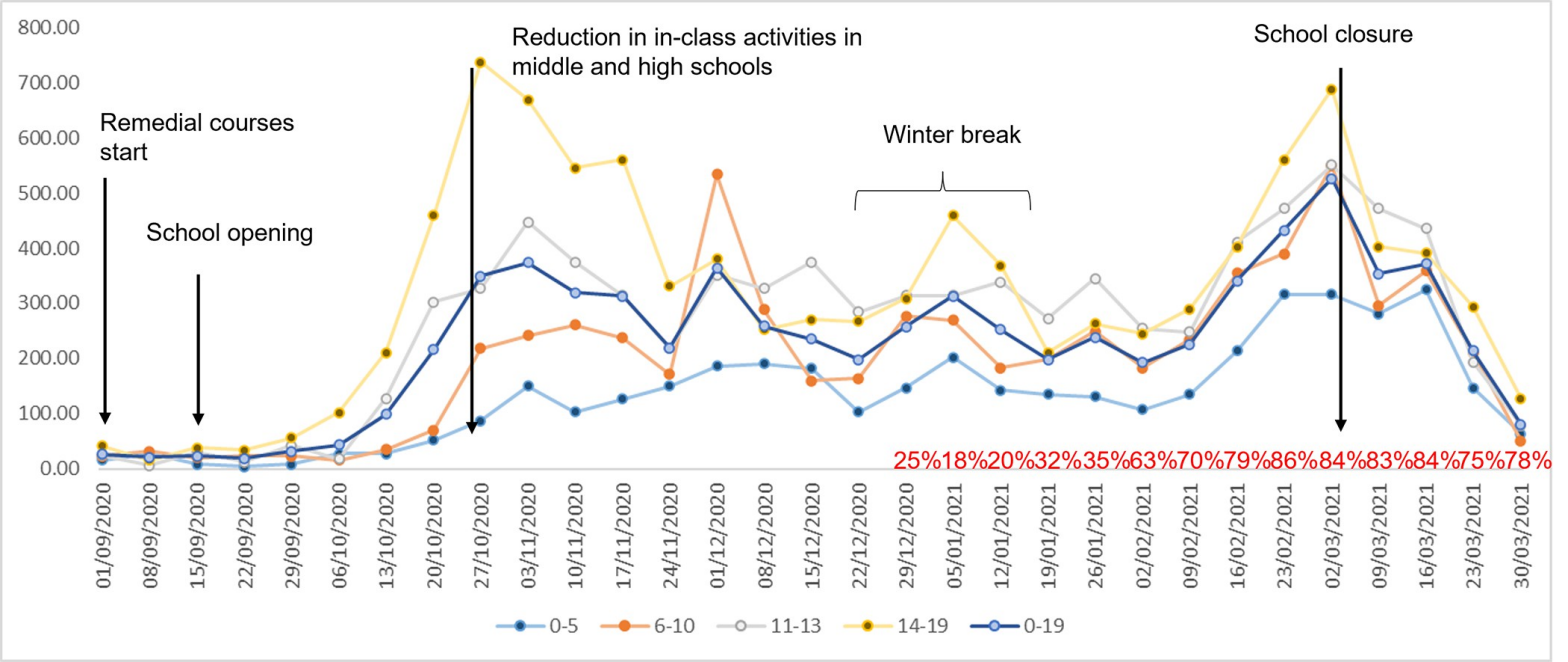
Weekly notification rates of new COVID-19 cases per 100,000 inhabitants aged 0–19 years, by age class, Reggio Emilia Province, 1 September 2020–31 March 2021. In the graph the main changes in school opening and closure are also reported, and (in red) the proportion of Alpha variant among sequenced cases as reported by the Italian National Institute of Health.

There are approximately 95,000 inhabitants in RE between the ages of six months and 19 years, commonly divided among the following levels: day care centres/nurseries (ages 0–3 years), preschools (ages 3–5), primary schools (ages 6–10), middle schools (ages 11–13) and high schools (ages 14–19). There are about 12,000 teachers/school staff members in the province (531,751 inhabitants, Emilia Romagna, Northern Italy). During the study period, there were two peaks of infections: in November 2020 and in February/March 2021 (Fig 2). After school reopening on 1 September 2020 for remedial courses and on 15 September 2020 for the regular school year, in-class learning was in place until 26 October 2020, when most high schools moved to remote learning for at least 75% of scheduled lessons. In addition, because of the significant circulation of the virus, the Christmas school holidays were extended to the second week of January, and thus from 20 December to 11/15 January. Another lockdown led to the closing of schools on 3 March 2021 (Fig 2). Only day care centres/nurseries and preschools, schools that required laboratory work and schools for pupils with disabilities or special needs continued in-class didactic activities.

### Design

An analysis of population-based surveillance data was carried out, including all consecutive positive cases confirmed by RT-PCR for SARS-COV-2 infection between 1 September 2020 and 4 April 2021 in Reggio Emilia that led to an epidemiological investigation among children and adolescents (0–19 years) who may have had exposure or contact with positive cases at school. Given that reporting positivity of even one child led to a field investigation, contact tracing and, in some cases, quarantine measures, all cases were included, regardless of whether an outbreak was present. We excluded cases that occurred among children not attending schools in the period investigated for contact tracing, namely in the period starting 48 hours before symptom onset, and for asymptomatic cases, 48 hours before diagnosis or 48 hours after contact with a certain case, whichever occurred first.

### Control measures

When a case occurred in primary and secondary schools, in-class activities were usually suspended but quarantine was not mandatory if all physical distancing measures were applied (sufficient distance in the classroom, physical distance during all activities and utilization of face masks). All students and school personnel were asked to perform a swab, with an appointment scheduled as early as possible and parents notified by means of a text message. If at least one secondary case was found, quarantine was mandatory for all students in that class. For all close contacts identified during the epidemiological investigation, a ten-day quarantine and a negative exit test were required. In day care centres/nurseries and preschool settings, both the teachers and children were considered close contacts if they had had contact with a case at school 48 hours before symptom onset or positive swab, in which case they were subjected to quarantine. In some cases, based on the surveillance team’s evaluation, the entire school was closed. Teachers were never quarantined for school contacts, except if they taught in a day care centres/nurseries or preschool.

The wearing of surgical masks was mandatory for students over the entire study period except when they were seated at their desks and not speaking, other than in day care centres/nurseries, where the use of masks was never mandatory. Other control measures have been described in more detail elsewhere [15]. The major changes occurring during the study period are described in Fig 1.

### Data sources

Following the identification and notification of a COVID-19 case, qualified Public Health Department (PHD) personnel performed detailed field investigations, managed the index case and identified contacts according to the regional recommendations and control measures in place. Comprehensive surveillance data containing information on index case, contacts, school and class characteristics, swabs performed, secondary cases and measures undertaken were collected by the PHD and stored in electronic forms. Each case and cluster was re-abstracted by a study investigator and checked for consistency and plausibility. Missing data were obtained from the COVID-19 Surveillance Registry software and a research database was constructed.

### Definition of index case and cluster

In this analysis we consider a class (each single case or outbreak) as one statistical unit. For each class, an index case was identified, defined as the first case who tested positive in a class (considering the date the swab was done). If more than one case in a class tested positive on the same day, the case with the earliest symptom onset was considered the index case. Presence of two or more positive students or teachers/staff members in the same class was defined as classroom cluster.

The same class may be included more than once because it may have been involved in more than one investigation during the study period, creating a school cluster. When more than one class was included in a between-class transmission or there was a single index case for more than one class (usually, but not only, when the index case was a teacher), this was considered a school cluster.

Index cases were classified as student or teacher/staff member and according to whether the case had a known contact outside the school setting (household, social activity, sports activity, not identified, and other/unspecified). The classes were classified according to the type of school (day care centres/nurseries and preschool, primary school, middle school, and high school) and whether they were part of a multiclass cluster.

### Outcome definition and variables of interest

The main outcomes were the secondary transmission rate and delay in diagnosis of the index case.

Given that a class was a unit of observation, secondary attack rates were calculated in two ways. The mean attack rate was calculated as the mean value of all class-level transmissions. Overall attack rates were calculated by dividing the number of cases by the population at risk, namely classmates, teachers and staff, who had had close contact with the index case in a period starting 48 hours before symptom onset of the symptomatic index case and, for asymptomatic cases, 48 hours before diagnosis or 48 hours after contact with a confirmed case, whichever occurred first.

If a classmate was already in isolation prior to symptom onset or swab positivity of the index case, due to contact with a positive person or re-entry from abroad, he/she was excluded from the denominator, as was any student or staff member who refused to perform a swab.

Delay in diagnosis of the index case was calculated (only for symptomatic cases) as the number of days between symptom onset and the date of swab positivity. Delay in contact tracing was calculated as the time from swab positivity of the index case to the date on which the swab for (the majority of) classmates was scheduled.

### Statistical analysis

Descriptive statistics were used to describe and summarize the data. We used Microsoft Excel and Stata v.13.1 (Statacorp, Tx) for data analysis. Class and cluster characteristics are reported. However, information about the characteristics of cases diagnosed outside the province or those that were identified during some experimental screening programmes (two classes) is missing. Ninety-five percent confidence intervals are reported for the overall attack rate based on binomial exact distribution and for the mean attack rate according to normal distribution. No tests of hypothesis were performed.

## Results

A total of 1,213 student and 391 teacher/staff index cases were identified, corresponding to 1,882 investigated classes, because some index cases–particularly teachers–had contacts in more than one class. A total of 43,792 swabs were requested for contact tracing in these classes and 43,214 (98.7%) were performed (Table 1). The number of students and teachers/staff members involved in contact tracing was slightly less than half of the total number of resident students and teachers/staff in the province. However, this does not mean that epidemiological investigations involved this proportion of the school population, because some classes might have been involved in more than one investigation during the study period. Teacher index cases were more frequent in preschools and primary schools than in secondary schools.

**Table 1:** Characteristics of the field investigation in 1,884* classes for which a school contact with a COVID-19 case was suspected, by type of index case. Reggio Emilia, September 2020-March 2021.

	Student		Teacher		Total*	
**Classes, n (%)** ^**&**^	**1,224**		**658**		**1,882**	
Type of school, n (%)						
Day care centre/nursery	194	15.9	156	23.7	350	18.6
Primary school	341	27.8	197	29.9	538	28.6
Middle school	310	25.3	186	28.3	496	26.3
High school	366	29.9	112	17.0	478	25.4
Other educational services	13	1.1	7	1.1	20	1.1
Secondary transmission, n (%)	471	38.5	255	38.7	726	38.6
Part of a school cluster, n (%)	105	8.6	412	62.6	517	27.5
School clusters, n (%)	51		142		193	
**Index cases (%)** ^**&**^	**1,213**	** **	**391**	** **	**1,604**	
Possible source of infection, n (%)						
Yes	686	56.6	104	26.6	790	49.2
No	527	43.4	287	73.4	814	50.8
Type of source of infection, n (%)						
Household outbreak	564	46.5	50	12.8	614	38.3
Social contact	19	1.6	7	1.8	26	1.6
Sport contact	18	1.5	0	0.0	18	1.1
Unidentifiable contact	85	7.0	47	12.0	132	8.2
**Field investigation results**						
Testing delay—mean days (SD)	6.1	4.8	4.8	4.2	5.6	4.6
Tracing delay—mean days (SD)	4.3	3.3	4.1	2.9	4.2	3.2
Number of contacts	30,184		13,608		43,792	
Number of tested contacts	29,802		13,412		43,214	
Number of secondary cases	1,047		658		1,705	
Overall attack rate, % (95% CI)^¥^ **	3.5	3.3–3.7	4.9	4.5–5.3	3.9	3.8–4.1
Mean attack rate^^^ **	3.6	3.2–4.1	5.0	4.2–5.8	4.1	3.7–4.5

n, number of students/staff; SD, standard deviation; CI, confidence interval.

* In two classes there were no index cases due to screening.

^&^ Percentages of the total number of classes or index cases.

** Swabs not performed were excluded, as were 11 classes with an unknown number of contacts.

^¥^ Calculated by dividing the overall number of secondary cases by the number of tested contacts.

^^^ Mean of all classroom-level attack rates.

Student index cases were identified in 56.5% of cases as a contact of other cases, the vast majority of which were within the same household (46.5%). A possible source of infection was identified for only 26.6% of teacher index cases (Table 1).

Secondary transmission occurred in fewer than 40% of classes, and the overall attack rate was 3.9% (95% CI 3.8%-4.1%), 3.5% (95% CI 3.3%-3.7%) for student index cases and 4.9% (95% CI 4.5%-5.3%) for teacher index cases (Table 1). The attack rate over the study period was 4.8% in September and October, 2.6% in November, 3.9% in December and 4% from January to April (Table 2). The attack rate was similar in all types of schools (Table 3).

**Table 2:** Characteristics of the field investigation in 1,884 classes for which a school contact with a COVID-19 cases was suspected, by calendar period. Reggio Emilia, September 2020-March 2021.

	September+ October		November		December		January+February+ March+April*	
**Classes, n (%)** ^**&**^	**248**		**263**		**316**		**1,057**	
Type of school, n (%)								
Day care centre/nursery	39	15.7	72	27.4	53	16.8	186	17.6
Primary school	46	18.6	89	33.8	113	35.8	292	27.6
Middle school	52	21.0	82	31.2	104	32.9	258	24.4
High school	111	44.8	18	6.8	42	13.3	307	29.1
Other educational services	0	0.0	2	0.8	4	1.3	14	1.3
Secondary transmission, n (%)	106	43.6	86	33.0	124	40.3	411	39.1
Part of a school cluster, n (%)	55	22.2	73	27.8	112	35.4	277	26.2
Number of school clusters	22		27		37		107	
**Index cases, n (%)** ^**&**^	**226**	** **	**220**	** **	**250**	** **	**908**	
Type of index case, n (%)								
Teacher	38	16.8	75	34.1	72	28.8	206	22.7
Student	188	83.2	145	65.9	178	71.2	702	77.3
Possible source of infection	100	44.2	54	24.5	138	55.2	498	54.8
Household outbreak	59	26.1	45	20.5	113	45.2	397	43.7
Social contact	7	3.1	0	0.0	5	2.0	14	1.5
Sport contact	7	3.1	0	0.0	0	0.0	11	1.2
Unidentifiable contact	27	11.9	9	4.1	20	8.0	76	8.4
No contact reported	126	55.8	166	75.5	112	44.8	410	45.2
**Field investigation results**								
Testing delay—mean days (SD)	5.7 (4.3)		5.8 (3.8)		7.0 (5.1)		5.2 (4.6)	
Tracing delay—mean days (SD)	7.4 (3.8)		7.4 (3.9)		3.6 (1.7)		2.9 (1.9)	
Number of contacts	6,327		6,279		6,860		24,314	
Number of tested contacts	6,252		5,964		6,827		24,204	
Number of secondary cases	302		156		264		984	
Overall attack rate, % (95% CI)^¥^ **	4.8	4.3–5.4	2.6	2.2–3.1	3.9	3.4–4.4	4.1	3.8–4.3
Mean attack rate^^^ **	4.8	3.8–5.9	2.8	2.0–3.5	4.2	3.2–5.2	4.2	3.7–4.8

n, number of students/staff; SD, standard deviation; CI, confidence interval.

*Until 4 April 2021.

^&^ Percentages of the total number of classes or index cases.

**Swabs not performed were excluded, as were 11 classes with an unknown number of contacts.

^¥^ Calculated by dividing overall secondary cases by the number of tested contacts.

^^^ Mean of all classroom-level attack rates.

**Table 3:** Characteristics of the field investigation in 1,884 classes for which a school contact with a Covid-19 cases was suspected, by type of school. Reggio Emilia, September 2020-March 2021.

	Day care centre/nursery		Primary school		Middle school		High school		Other educational services	

**Classes, n (%)** ^**&**^	**350**		**540**		**496**		**478**		**20**	
Secondary transmission, n (%)	131	37.4	216	40.0	173	34.9	202	42.3	5	25.0
Part of a school cluster, n (%)	25	7.1	162	30.0	207	41.7	116	24.3	7	35.0
Number of school clusters	10		70		68		43		2	
**Index cases, n (%)** ^**&**^	**338**		**466**		**370**		**415**		**15**	
Type of index case, n (%)										
Teacher	144	42.6	126	27.0	66	17.8	51	12.3	4	26.7
Student	194	57.4	340	73.0	304	82.2	364	87.7	11	73.3
**Field investigation results**										
Number of contacts	7,767		11,709		12,106		11,936		306	
Number of tested contacts	7,652		11,575		11,923		11,799		298	
Number of secondary cases	349		553		386		409		9	
Overall attack rate, % (95% CI)^¥^ *	4.6	4.1–5.1	4.8	4.4–5.2	3.2	2.9–3.6	3.5	3.1–3.8	3.0	1.4–5.7
Mean attack rate^^^ *	4.8	3.7–5.9	5.0	4.1–5.9	3.4	2.8–4.0	3.3	2.8–3.9	2.1	0.3–4.5
Testing delay—mean days (SD)	6.0	4.6	5.8	4.9	5.4	4.6	5.4	4.3	6.7	5.1
Tracing delay—mean days (SD)	4.4	3.5	4.1	3.1	4.1	3.3	4.4	3.0	3.3	2.3

n, number of students/staff; SD, standard deviation; CI, confidence interval.

^&^ Percentages of the total number of classes or index cases.

*Swabs not performed were excluded, as were 11 classes with an unknown number of contacts.

^¥^ Calculated by dividing the overall number of secondary cases by the number of tested contacts.

^^^ Mean of all classroom-level attack rates.

The mean delay in testing was 6.0 days in student index cases and 4.8 for teachers (Table 1). This did not change over the study period, except for a slight increase in December, and was similar across all types of schools (Table 3). Conversely, the delay in contact tracing decreased from December 2020 onwards, from about seven days to three days (Table 2), although no differences can be seen in terms of type of index case and type of school (Table 3).

## Discussion

We can confirm a modest secondary attack rate in schools, as already observed in previous studies [17–20]. Secondary cases occurred in about 40% of classes that were exposed to an infected classmate or teacher, and the overall attack rate was about 4%. The attack rate was similar during the study period, despite an expected increase in the secondary case attack rate starting in late December/January in the region due to the spread of the Alpha variant [16] which is more infectious than the previously circulating variants [21], and despite that control measures were put in place in early November, including the mandatory use of face masks for children over the age of six and reduced in-presence attendance, especially in high and middle schools.

Unlike the findings in our previous report [15] and other studies reporting data on school cluster investigations [20], the attack rate was similar in preschools, primary schools and secondary schools. In fact, compared with the first two months of the study period [15], the attack rate increased in preschools from almost no transmission to 4%, while it remained stable in secondary schools despite that remote learning and periodic closures were implemented much more frequently in secondary schools, starting from 26 October 2020. The possible reason for similar rates over time in secondary schools regardless of the implemented measures might be a high level of out-of-school contact in secondary schools. Furthermore, preschools were the only setting where face masks were not used by children during the whole period.

Surprisingly, the difference in attack rate between classes where the index case was a student and those where the index case was a teacher was also small, despite the different kinds of interactions and length of exposure of student-student contact and teacher-student contact in the school setting. The attack rate was only slightly higher in classes in which a teacher was an index case. It is worth noting that teacher index cases were more frequent in day care centres/nurseries and primary schools where children did not wear masks and where teachers had closer contact with children. Unfortunately, we do not have any analytical information making it possible to establish the quality and duration of interactions between index cases and contacts.

The effect of changes in contact tracing strategies introduced primarily after the second wave of the pandemic (thus at the end of November 2020) was appreciable in terms of the reduction in the delay in contact tracing and the increase in the proportion of index cases for which a contact was known, particularly a contact within the household. Indeed, a higher proportion of school investigations with a known link to household clusters was specifically the anticipated effect of the introduction of immediate testing of all asymptomatic contacts for all incident cases in the community, because this backward tracing made it possible to identify asymptomatic or paucisymptomatic students and to link them to their school contacts.

The main limitation of this study is that any observed association between changes in secondary attack rate and school or index case characteristics, and especially actions implemented during the study period, could be confounded by other factors that we could not measure. Among these, the most important was the spread of the Alpha variant, which probably started in the region around Christmas and which became the dominant circulating variant in February. Unfortunately, as very few cases were sequenced in Italy in that period [16], we cannot determine which clusters were caused by the Alpha variant.

Furthermore, we do not have any meaningful information about the measures put in place at single cluster or class level. In fact, it was impossible to establish when exactly the actions were put in place and many of these measures were actually introduced after the field investigation was carried out, which means that only reverse causality could be observed.

Other variables that were missing or difficult to standardize were the duration and intensity of contacts between the index case and other classmates and the control measures each school put in place independently both before the occurrence of the case and during the outbreak. Finally, it is impossible to transfer any insight from this experience to the current situation with the omicron variant, where contact tracing is not active, control measures and personal behaviours have been relaxed, and transmissibility has increased.

The main strength of the study is its population-based nature–it included all cases for which a school contact or exposure was identified. Despite all the limitations of the study design, it is worth reporting an analysis of routine surveillance data with a surprisingly high level of completeness of field investigations and test compliance. Only a few classes were not characterized, and very few classmates refused to be tested. Each outbreak in each school was investigated and re-checked for plausibility by the investigation team, thereby partially overcoming the shortcomings of retrospective and self-reported studies.

## Conclusions

Despite the increase in incidence during the autumn and the spread of a highly transmissible new variant, secondary transmission in schools did not increase during the period. Contact tracing improved, as suggested by the shorter delay in investigation and more frequent identification of the possible source of infection of the index case.

## Supporting information

S1 Table Number of classes, contacts, tested contacts and secondary cases by characteristics of classes, index cases and investigation. (DOCX)

## References

[1] Our World in Data. Cumulative confirmed COVID-19 cases. Available at: https://ourworldindata.org/explorers/coronavirus-data-explorer.

[2] COVID-19 Cumulative Infection Collaborators. Estimating global, regional, and national daily and cumulative infections with SARS-CoV-2 through Nov 14, 2021: a statistical analysis. Lancet. 2022;399(10344):2351–2380. doi: 10.1016/S0140-6736(22)00484-6 35405084 PMC8993157

[3] Viner RM, Mytton OT, Bonnel C, Mellendez-Torres JG, Ward J, Hudson L, et al. Susceptibility to SARS-CoV-2 Infection Among Children and Adolescents Compared With Adults. A Systematic Review and Meta-analysis. JAMA Pediatr. 2021;175(2):143–156. 32975552 10.1001/jamapediatrics.2020.4573 PMC7519436

[4] Gaythorpe KAM, Bhatia S, Mangal T, Unwin HJT, Imai N, Cuomo-Dannenburg G, et al. Children’s role in the COVID-19 pandemic: a systematic review of early surveillance data on susceptibility, severity, and transmissibility. Sci Rep. 2021 Jul 6;11(1):13903. doi: 10.1038/s41598-021-92500-9 34230530 PMC8260804

[5] Haug N, Geyrhofer L, Londei A, Dervic E, Desvars-Larrive A, Loreto V, et al. Ranking the effectiveness of worldwide COVID-19 government interventions. Nat Hum Behav. 2020;4(12):1303–1312. doi: 10.1038/s41562-020-01009-0 33199859

[6] Head JR, Andrejko K, Cheng Q, Collender PA, Phillips S, Boser A, et al. The effect of school closures and reopening strategies on COVID-19 infection dynamics in the San Francisco Bay Area: a cross-sectional survey and modeling analysis. medRxiv [Preprint]. 2020. doi: 10.1101/2020.08.06.20169797 32793934 PMC7418765

[7] Keeling MJ, Tildesley MJ, Atkins BD, Penman B, Southall E, Guyver-Fletcher G, et al. The impact of school reopening on the spread of COVID-19 in England. Philos Trans R Soc Lond B Biol Sci. 2021;376(1829):20200261. doi: 10.1098/rstb.2020.0261 34053259 PMC8165595

[8] Landeros A, Ji X, Lange K, Stutz TC, Xu J, Sehl ME, et al. An examination of school reopening strategies during the SARS-CoV-2 pandemic. PLoS One. 2021;16(5):e0251242. doi: 10.1371/journal.pone.0251242 34014947 PMC8136712

[9] Walsh S, Chowdhury A, Russell S, Birch JM, Ward JL, Waddington C, et al. Do school closures reduce community transmission of COVID-19? A systematic review of observational studies. BMJ Open. 2021;11(8):e053371. 10.1136/bmjopen-2021-053371 PMC8375447 34404718

[10] Courtemanche C, Garuccio J, Le A, Pinkston J, Yelowitz A. Strong Social Distancing Measures In The United States Reduced The COVID-19 Growth Rate. Health Aff (Millwood) 2020; 39: 1237–46. doi: 10.1377/hlthaff.2020.00608 32407171

[11] Hsiang S, Allen D, Annan-Phan S, Bell K, Bolliger I, Chong T, et al. The effect of large-scale anti-contagion policies on the COVID-19 pandemic. Nature 2020; 584: 262–7. doi: 10.1038/s41586-020-2404-8 32512578

[12] Ertem Z, Schechter-Perkins E, Oster E, van den Berg P, Epshtein I, Chaiyakunapruk N, et al. The Impact of School Opening Model on SARS-CoV-2 Community Incidence and Mortality: A Nationwide Cohort Study. Res Sq [Preprint]. 2021 Jul 15:rs.3.rs–712725. doi: 10.21203/rs.3.rs-712725/v1 34282412 PMC8288150

[13] Forbes H, Morton CE, Bacon S, McDonald HI, Minassian C, Brown JP, et al. Association between living with children and outcomes from covid-19: OpenSAFELY cohort study of 12 million adults in England. BMJ. 2021;372:n628. doi: 10.1136/bmj.n628 33737413 PMC7970340

[14] Marziano V, Guzzetta G, Rondinone BM, Boccuni F, Riccardo F, Bella A, et al. Retrospective analysis of the Italian exit strategy from COVID-19 lockdown. Proc Natl Acad Sci U S A. 2021;118(4):e2019617118. doi: 10.1073/pnas.2019617118 33414277 PMC7848712

[15] Larosa E, Djuric O, Cassinadri M, Cilloni S, Bisaccia E, Vicentini M, et al. Secondary transmission of COVID-19 in preschool and school settings in northern Italy after their reopening in September 2020: a population-based study. Euro Surveill. 2020;25(49):2001911. doi: 10.2807/1560-7917.ES.2020.25.49.2001911 33303065 PMC7730487

[16] Istituto Superiore di Sanità. Prevalenza e distribuzione delle varianti di SARS-CoV-2 di interesse per la sanità pubblica in Italia. Rapporto n. 5 del 23 luglio 2021. July 2021. [Italian] Available at: https://www.epicentro.iss.it/coronavirus/pdf/sars-cov-2-monitoraggio-varianti-rapporti-periodici-23-luglio-2021.pdf.

[17] Ehrhardt J, Ekinci A, Krehl H, Meincke M, Finci I, Klein J, et al. Transmission of SARS-CoV-2 in children aged 0 to 19 years in childcare facilities and schools after their reopening in May 2020, Baden-Württemberg, Germany. Euro Surveill. 2020;25(36):2001587. 10.2807/1560-7917.ES.2020.25.36.2001587 PMC7502898 32914746

[18] Macartney K, Quinn HE, Pillsbury AJ, Koirala A, Deng L, Winkler N, et al. Transmission of SARS-CoV-2 in Australian educational settings: a prospective cohort study. Lancet Child Adolesc Health. 2020;4(11):807–816. doi: 10.1016/S2352-4642(20)30251-0 32758454 PMC7398658

[19] Yoon Y, Kim KR, Park H, Kim S, Kim YJ. Stepwise school opening online and off-line and an impact on the epidemiology of COVID-19 in the pediatric population. J Korean Med Sci. 2020 Nov 30;35(46):e414. 33258334 10.3346/jkms.2020.35.e414 PMC7707922

[20] Aiano F, Mensah AA, McOwat K, Obi C, Vusirikala A, Powell AA, et al. COVID-19 outbreaks following full reopening of primary and secondary schools in England: Cross-sectional national surveillance, November 2020. Lancet Reg Health Eur. 2021;6:100120. doi: 10.1016/j.lanepe.2021.100120 34278370 PMC8276523

[21] Davies NG, Abbott S, Barnard RC, Jarvis CI, Kucharski AJ, Munday JD, et al. Estimated transmissibility and impact of SARS-CoV-2 lineage B.1.1.7 in England. Science. 2021;372(6538):eabg3055. doi: 10.1126/science.abg3055 33658326 PMC8128288

