# Supplementary material for: Surveillance, contact tracing and characteristics of SARS-CoV-2 transmission in educational settings in Northern Italy, September 2020 to April 2021
Source: PLoS One. 2022 Oct 10;17(10):e0275667. doi: 10.1371/journal.pone.0275667 (PMC9550042; doi:10.1371/journal.pone.0275667)
Supplement: S1 Table — (DOCX) [file pone.0275667.s001.docx]

**S1 Table: Number of classes, contacts, tested contacts and secondary cases by characteristics of classes, index cases and investigation**

|  | **No of classes** | **No of contacts** | **No of tested contacts** | **No of secondary cases** |
| --- | --- | --- | --- | --- |
| **Classes** |  |  |  |  |
| Type of school |  |  |  |  |
| Day care centre/nursery | 350 | 7,768 | 7,652 | 349 |
| Primary school | 540 | 11,709 | 11,575 | 553 |
| Middle school | 496 | 12,106 | 11,923 | 386 |
| High school | 478 | 11,936 | 11,799 | 409 |
| Other educational services | 20 | 306 | 298 | 9 |
| Secondary transmission |  |  |  |  |
| Yes | 727 | 26,245 | 25,766 | 1,706 |
| No | 1,157 | 17,580 | 17,481 | 0 |
| Part of a school cluster |  |  |  |  |
| Yes | 517 | 10,983 | 10,863 | 435 |
| No | 1,367 | 32,842 | 32,384 | 1,271 |
| **Index cases** |  |  |  |  |
| Type of index case |  |  |  |  |
| Teacher | 658 | 13,608 | 13,412 | 658 |
| Student | 1,224 | 30,184 | 29,802 | 1,047 |
| Screening | 2 | 33 | 33 | 1 |
| Possible source of infection |  |  |  |  |
| Yes | 790 | 19,952 | 19,805 | 646 |
| No | 1,094 | 23,873 | 23,442 | 1,060 |
| Type of source of infection |  |  |  |  |
| Household outbreak | 614 | 15,445 | 15,324 | 490 |
| Social contact | 26 | 683 | 679 | 20 |
| Sport contact | 18 | 497 | 493 | 15 |
| Unidentifiable contact | 132 | 3,327 | 3,309 | 121 |
| **Field investigation results** |  |  |  |  |
| Calendar period |  |  |  |  |
| September+October | 248 | 6,327 | 6,252 | 302 |
| November | 263 | 6,279 | 5,964 | 156 |
| December | 316 | 6,860 | 6,827 | 264 |
| January-April* | 1,057 | 24,314 | 24,204 | 987 |
